# Kounis syndrome secondary to nimesulide ingestion: a case report

**DOI:** 10.1186/s43044-021-00233-x

**Published:** 2021-12-20

**Authors:** Yamasandi Siddegowda Shrimanth, Krishna Santosh Vemuri, Atit A. Gawalkar, Soumitra Ghosh, Jyothi Vijay, Thammannagowda Prarthana, Bhupendra Kumar Sihag

**Affiliations:** 1 Department of Cardiology, Post Graduate Institute of Medical Education and Research, Chandigarh, 160012 India; 2 Department of Dermatology Venereology Leprology, Post Graduate Institute of Medical Education and Research, Chandigarh, 160012 India

**Keywords:** Allergic reaction, Nimesulide, Kounis syndrome, ST-segment elevation myocardial infarction, Percutaneous coronary intervention

## Abstract

**Background:**

Kounis syndrome, also known as "allergic myocardial infarction," is a rare co-occurrence of acute coronary syndrome (ACS) in the setting of hypersensitivity reaction to any agent. Non-steroidal anti-inflammatory drugs (NSAIDs) like are often implicated in causing allergic reactions. Here, we present a case of anterior wall myocardial infarction (AWMI) occurred following angioedema secondary to intake of Nimesulide, not described earlier in literature.

**Case presentation:**

A 45-year-old female developed generalized pruritic, erythematous maculopapular rash, facial puffiness, oral ulcers and hoarseness of voice within few hours following consumption of Nimesulide for fever and body-ache. Due to development of hypotension, electrocardiogram (ECG) was done, which revealed ST elevation in V2–V6, with marked elevation of troponin (TnI) and creatine kinase (CK-MB). He had no chest pain or shortness of breath. Echocardiography showed regional wall motion (RWMA) abnormality in left anterior descending artery (LAD) territory with an ejection fraction of 25%. Coronary angiography showed a complete thrombotic cutoff of LAD, for which Tirofiban infusion was started to decrease thrombus burden. Repeat angiography on next day showed 80% lesion in proximal LAD for which she underwent revascularization with a drug-eluting stent. The patient later showed improvement in cardiac function at 8 months of follow-up.

**Conclusions:**

The occurrence of ACS requiring percutaneous coronary intervention (PCI) in the setting of allergic reactions is rarely reported in the literature. One should be aware of the rare possibility of Kounis syndrome in the setting of hypersensitivity reaction when accompanying features of symptoms suggestive of coronary artery disease co-exists. When indicated, ECG monitoring and cardiac biomarkers in patients with allergic responses help to identify this rare and treatable condition.

## Background

Kounis syndrome (KS) is a coincidental occurrence of acute coronary syndrome (ACS) and hypersensitivity reactions following an allergic reaction involving the release of inflammatory cytokines with mast cell and platelet activation leading to coronary artery vasospasm and/or atheromatous plaque erosion or rupture [1, 2]. It was first described in 1991 by Kounis and Zavras as "allergic angina syndrome" or "allergic myocardial infarction" [3]. It occurs secondary to allergic insults from food, drugs, insect bites. Non-steroidal anti-inflammatory drugs (NSAIDs) like Aspirin and Nimesulide are also often implicated in causing allergic reactions. Herein, we present a case of anterior wall myocardial infarction (AWMI) in a middle-aged female that occurred following angioedema secondary to intake of Nimesulide, not described earlier in literature.

## Case presentation

A 45-year-old hypertensive, hypothyroid (on 100 mcg thyroxine) female presented with fever and non-specific joint-ache and body-ache for 2 days, for which she took over the counter analgesic Nimesulide. Following the drug intake, she developed a generalized erythematous maculopapular rash associated with pruritis within 2–3 h, which started on the face and rapidly involved whole body. Within few hours she also developed facial puffiness and oral ulcers. It was associated with gradual development of hoarseness of voice simultaneously. Due to the worsening of symptoms, she visited a local hospital within 4 h of symptom onset. At admission there, she had blood pressure of 90/60 mm hg, heart rate of 112 per min, and temperature of 99°F and saturation of 88% on room air. Clinical Examination showed bilaterally symmetrical non-pitting peri-orbital swelling involving both upper and lower eyelids along with lip swelling and generalized erythema suggestive of angioedema She also had diffuse bilateral wheezing. She did not have any chest pain or shortness of breath. She was stabilized intravenous hydrocortisone 100 mg and diphenhydramine 50 mg and fluids, oxygen supplementation, and referred to our center.

On admission to our center on the same day, her blood pressure of 100/70 mm Hg, pulse rate was 100/minute. According to her attendant peri-orbital and generalized swelling and erythema were improved than before. We continued with steroid, antihistaminic, oxygen and other supportive measures. Routine blood examination revealed hemoglobin of 12.5, total leukoyte count of 8200 with eosinophil of 18%. Urea and creatinine were 98 mg/dL and 2.1 mg/dL most likely suggestive of pre-renal acute kidney injury due to hypotension.

**Fig. 1:**
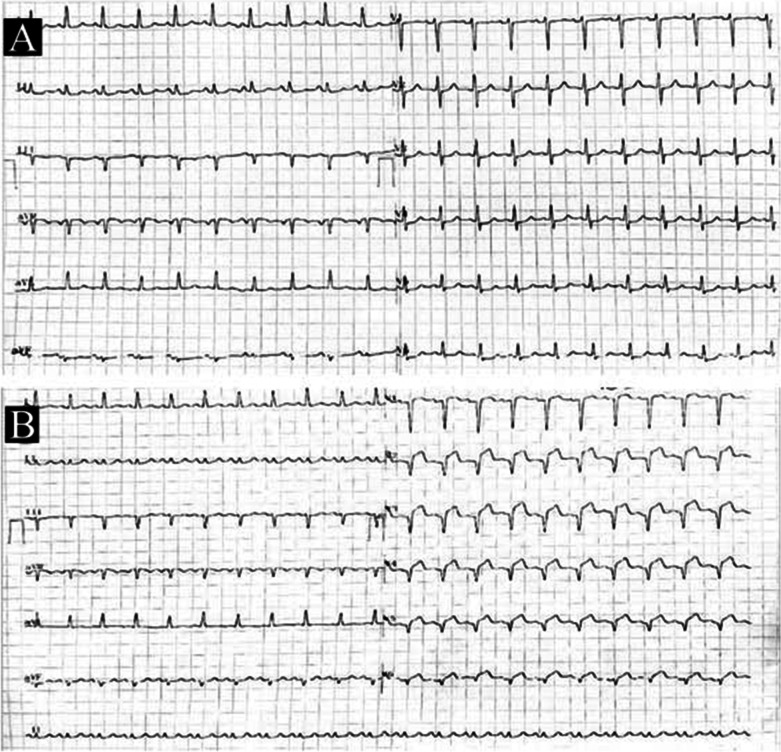
Panel **A** Electrocardiogram at baseline showing sinus rhythm with left axis deviation and mild ST depressions in V4–V6 leads. Panel **B** ECG showing ST elevations in anterolateral leads

**Fig. 2:**
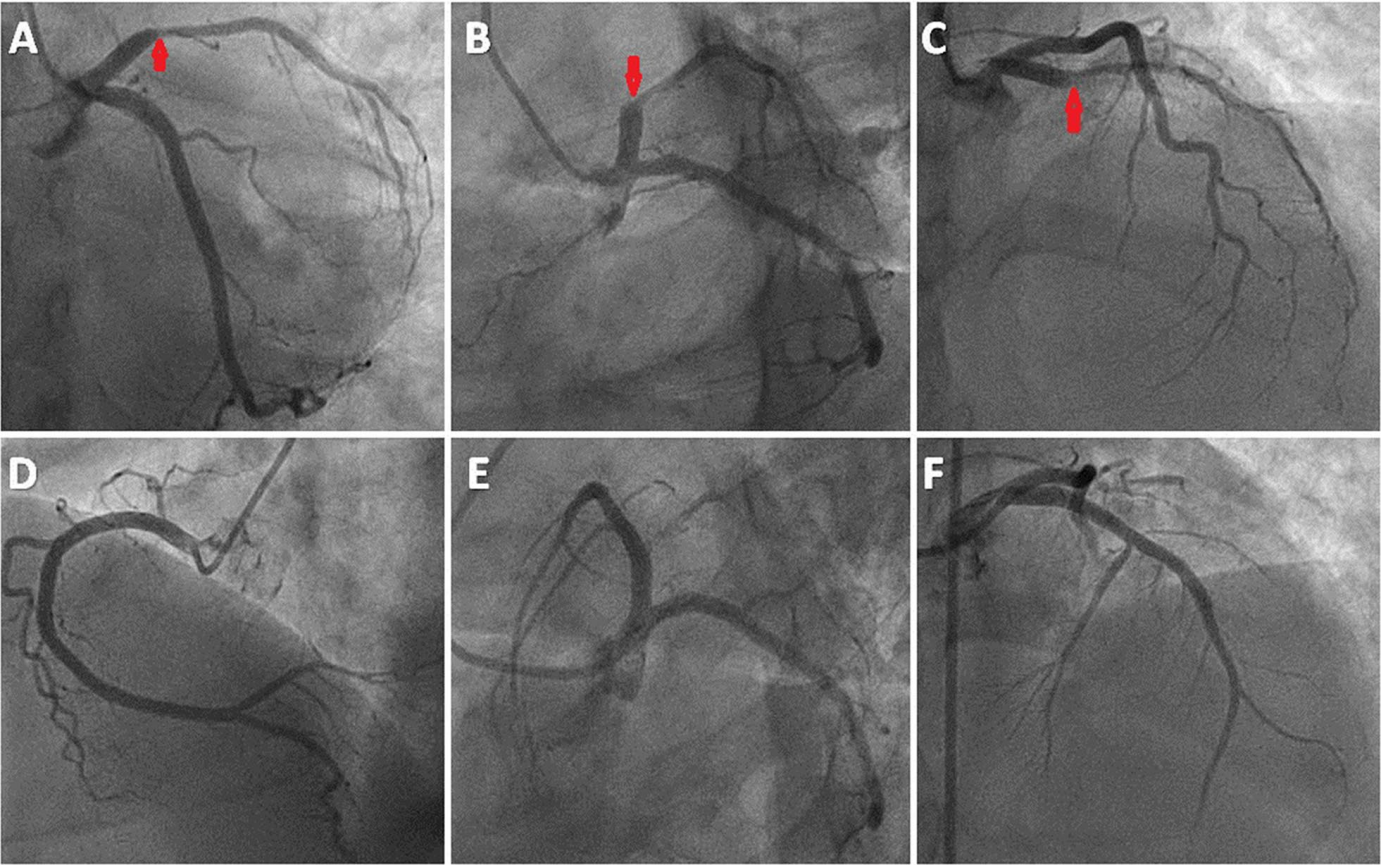
Coronary angiography (Panel **A**–**D**) showing 100% occlusion of proximal LAD (red arrow) and normal LCX and RCA. Coronary angiography (Panel **E**, **F**) after DES implantation to LAD with TIMI3 flow in AP cranial and LAO Caudal views

Next day early morning, during ICU stay, her BP fell down to 80/50 for which norepinephrine had to be started. To reveal the cause of hypotension, electrocardiogram (ECG) was done, which showed new onset ST-segment elevation in V2–V6 (Fig. 1A, B). High sensitivity cardiac troponin T (hs-cTnT) and creatine kinase (CK-MB) were elevated to 25.3 ng/ml (normal: 0–0.4 ng/ml) and 254 ng/ml (normal: 0–4.3 ng/ml), respectively. Echocardiography showed regional wall motion abnormalities (RWMA) in left anterior descending (LAD) artery territory with an ejection fraction of 25%. A diagnosis of silent anterior wall myocardial infarction (AWMI) was made and she was started on dual antiplatelets, high-intensity statin, low molecular weight heparin (renal modified dosage) and was taken up for emergency angiography. Coronary angiography showed a complete cut off of the LAD with a heavy thrombotic burden and normal other coronaries (Fig. 2A–D). Percutaneous coronary intervention (PCI) was abandoned in view of high thrombus load and patient was started on Tirofiban infusion to decrease thrombus burden. Plan was to do a check angiography later after Tirofiban infusion. Repeat angiography showed 80% lesion in proximal LAD with some thrombus near it and thrombotic cut off of mid main diagonal branch (D1). Diagnosis of type 2 Kounis syndrome was made in view of allergic manifestations along with presence of ruptured or erosed coronary plague leading to thrombus formation resulting in acute MI. PCI to LAD was done with drug-eluting stent 3.5 × 38 mm and balloon angioplasty (POBA) was done to D1 successfully with thrombolysis in myocardial infarction 3 (TIMI 3) flow (Fig. 2E, F).

She subsequently developed worsening of acute kidney injury with elevated serum creatinine level up to 4 mg/dl most likely due to hypotension, which improved in a week to normal value. Immunological work was sent to rule out connective tissue disorders, which was negative. She improved symptomatically, her rash disappeared, and swelling subsided and was discharged on day ten with precautionary advice against the usage of nimesulide. The patient is under regular follow-up for the last 8 months and her ejection fraction improved to 55%.

## Discussion

Allergic reactions in the cardiac critical care unit are mostly due to either contrast mediated or drugs used for sedation. But allergic reaction predisposing the individual to develop acute coronary syndrome is rare and is reported in the literature. Non-steroidal anti-inflammatory drugs (NSAIDs) are one of the drugs implicated in causing anaphylactic reactions. There were case reports of Kounis syndrome secondary to Aspirin [4] and Diclofenac [5]. Kounis syndrome (KS) is defined as acute coronary syndrome due to an allergic insult. The majority of the cases (80%) occur within 1 h of exposure to the trigger. It is mediated by inflammatory mediators like histamine, neutral proteases chymase, tryptase, and cathepsin D, which increases the production of leukotrienes [6]. Three variants of KS were described, with type 1 being most common, which accounts for 73%, where ACS is due to spasm of the coronary artery without underlying atherosclerosis. Type 2 (22%) occurs in patients with pre-existing but asymptomatic coronary artery disease in which ACS is due to plaque erosion or rupture of plaque. Type 3 (5%) represents stent thrombosis due to an allergic reaction. The risk factors include smoking, diabetes, hypertension, prior allergies, and dyslipidemia [1]. Intra-coronary imaging like intra vascular ultrasound (IVUS) and optical coherence tomography (OCT) has potential role in determining type of type of Kounis syndrome and its management. They can detect vasospam, plaque erosion or rupture and stent thrombosis [7] The management of KS patients includes treatment of both allergic reactions and acute coronary syndrome, which is complex as the drugs used in the treatment of anaphylaxis like epinephrine may worsen coronary artery spasm, prolongs QT interval, and precipitate arrhythmias and the fluids for the management of distributive shock in anaphylaxis may cause worsening of pulmonary edema, and the glucocorticoids may cause impairment of wound healing causing myocardial thinning, cardiac aneurysms and cardiac free wall rupture [2, 8]. The management of patients with type 1 KS is the treatment of allergic events and medical management of ACS. Whereas in type 2 (our case) and type 3 KS, the management includes PCI, which needs to be done in time. In our case, even though earlier ECG showed normal sinus rhythm and tachycardia, repeat ECG at the time of admission showed anterior wall myocardial infarction (AWMI). Once the diagnosis of AWMI was made, she was taken up for angiography. Because of the heavy thrombus burden, she was initially managed with tirofiban infusion followed by stenting [9]. Our case differs from the rest in that the patient did not have chest pain and was diagnosed based on ECG done for shock and shortness of breath at the time of admission. The occurrence of acute kidney injury secondary to allergic reaction prolonged the hospital stay in our case. This case highlights the importance of doing an ECG in all patients presenting with allergic reactions and also the pivotal role of follow-up ECG to rule out Kounis syndrome, as our index case had no chest pain at the time of presentation and also after admission.

## Conclusions

The occurrence of ACS requiring percutaneous intervention in the setting of allergic reactions, even though rare, has been reported in the literature. Our case is a rare case of Nimesulide induced Kounis syndrome, which was successfully managed. ECG monitoring and cardiac biomarkers in all patients with severe allergic reactions to identify this rare and treatable condition are reasonable.

## Data Availability

Data sharing is not applicable to this article as no datasets were generated or analyzed during the current study.

## Abbreviations

ACS: Acute coronary syndrome
AWMI: Anterior wall myocardial infarction
CK-MB: Creatine kinase MB fraction
ECG: Electrocardiogram
KS: Kounis syndrome
LAD: Left anterior descending
NSAIDS: Non-steroidal anti-inflammatory drugs
PCI: Percutaneous coronary intervention
TIMI: Thrombolysis in myocardial infarction
